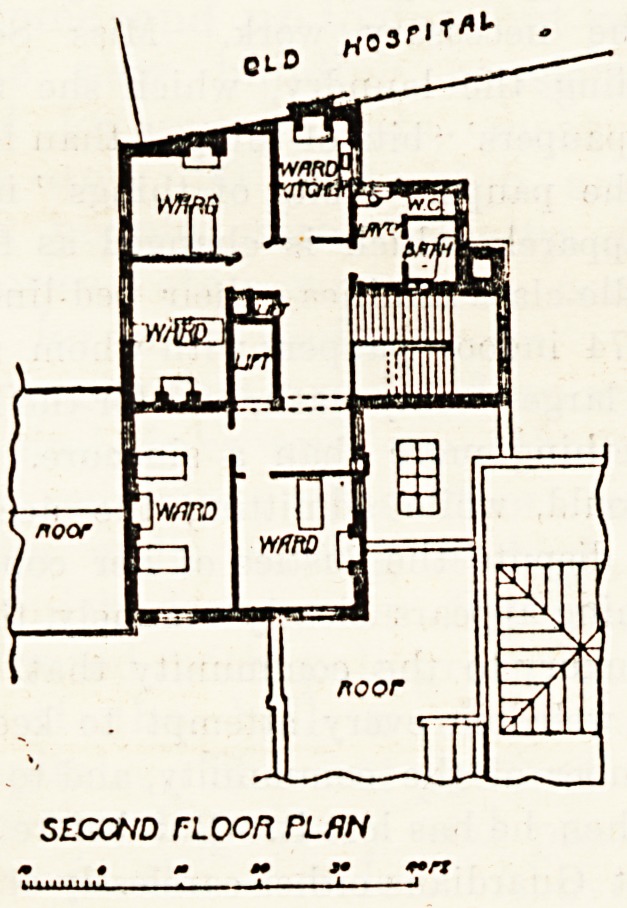# New Hospital for Women and Children, Leeds

**Published:** 1905-09-09

**Authors:** 


### NEW HOSPITAL FOR WOMEN AND CHILDREN, LEEDS.

The old hospital dates as far back as 1853, in which year a house in East Parade was opened for the reception of patients, and in 1855 it was enlarged by taking in part of an adjoining house. In 1860 the Springfield Lodge Estate of 6,500 square yards was bought and the private residence standing thereon was converted into a hospital, the total expense being £5,000. This arrangement was made to answer for a time, but it was soon felt that more adequate accommodation would be needed, and this necessity cannot be questioned when we learn that in the year 1902 the old hospital had treated 390 in-patients, 3,925 out-patients, and that 385 operations were performed.

At the annual meeting in 1896, a hope was expressed by the managers that a new hospital would be erected, and a nucleus for the building fund was provided by the handsome donation of £4,000 from Colonel Gasgoine of Parlington. In 1898 plans were prepared for gynæcological wards for 50 patients and for a maternity department for 16 beds. The estimated expense was £33,000. This entailed a larger expenditure than the committee felt justified in incurring, but an opportune gift of £8,000 from Mr. Arthington brought the building fund up to nearly £15,000. This sum was deemed sufficient for a beginning of operations, and the building scheme was revised, after which new plans were drawn up and approved; the work was proceeded with, and the new hospital was opened in the year 1903, but the maternity department was not erected. Pending further action in this, a separate portion of the building has been efficiently equipped, and will answer extremely well for a few lying in cases.

The old hospital has been utilised as an administrative department, and in it are accommodated the matron, the nursing, and the domestic staffs. The kitchen and store departments are also there.

Adjoining the old building are the new board-room and the secretary's office, and the passage of communication gives access also to the staircase, the lift, the lavatory for the staff, and to the hall; along the east side of the latter are the admission-room, the porch, and the hall-porter's room. To the north are the dispensary and the whole of the out-patients' department with its separate entrance and exit. At the south end of the hall is the entrance to the ward block or ward unit. There is a corridor about 38 feet long, on the east side of which are a three-bedded room and a single-bedded room, and on the west side are the lavatory, ba h-room, linen-rooms, and ward-kitchen. The corridor can hardly fail to be somewhat dark. The three-bedded room is provided with a large bay window, but the single-bedded room has only one window. Supposing these rooms to have fanlights over the doors they will obtain some cross ventilation thereby, but at best it will be only into a corridor, hence is unlikely to be quite satisfactory. Both rooms have open fire-places. At the south end of the corridor is the large ward, which has its long diameter correctly placed north and south. The ward contains 20 beds, is 100 feet long, 24 feet wide, and 13 feet high. Each bed has 10 feet of wall space, 120 square feet of floor space, and 1,565 feet of cubic space; and all these dimensions must be pronounced quite satisfactory. The ward has a large bay window at its south end, and another on the west side, and, excepting the two beds at the north end, each bed has a window on both sides. At the south-east corner is the sanitary annexe containing lavatory sink, and two w.c.'s. A fire escape staircase is incorporated. It is pointed out in the descriptive letterpress sent to us, that as only one of the usual sanitary wings is present, the south end and the south-west angle are free, and consequently more sun and light will enter at the south aspect of the ward. In a climate like that of England there is a good deal to be said in favour of this arrangement, but the objections strike one at once that unless the annexe be very large the lavatories are likely to be in close proximity to the closets, and that the bath-room has to form part of the main block which are certainly drawbacks. Still, the arrangement is one that will have its advocates. The ward floors are laid with terrazzo; the walls are finished in silicon plaster, and are enamelled with Ripoline. As far as possible all projections have been rounded off, and the angles have been replaced by coves. The window surface is large, but the windows are double, and in alternate windows the space between the outer and the inner has a steam coil placed in it. The warming is by open fire-places assisted by steam radiators.

The operation-room is placed at the north-east corner of the first-floor landing. It comprises a cloak-room, an anæsthetic-room, a recovery-room, and the theatre, which is 24 feet by 24 feet. It is extremely well lighted, and is heated by steam-coils, and there is an extraction shaft for getting rid of the foul air. The floor is of terrazzo, and it slopes slightly towards the north, so that it can be easily flushed, and hydrants for this purpose are provided. The walls of the theatre are finished in the same manner as those of the wards.

SECOND FLOOR PLAN

The first-floor ward is identical with the ground-floor one. The maternity wards, four in number and containing five beds in all, are on the second floor, and they occupy a space on this floor which pretty nearly corresponds to that occupied on the first floor by the resident medical officers' rooms, the landing, and the hall-porter's bedroom. This department constitutes a special feature of the hospital. Hitherto there has not been in Leeds any hospital accommodation for lying-in patients save what is provided at the workhouse. It is hoped that ultimately sufficient funds will be forthcoming to establish a lying-in hospital on a larger scale.

A general review of these plans gives a distinctly favourable impression. The large majority of the planning and the arrangements are so good that it is a pity the blemishes, which we felt bound to notice, exist. Another point is that infectious disease is almost sure to make its appearance sooner or later; and, beyond a very small isolation room in the out-patients' department, there is no isolation room in the hospital so far as the plans sent to us are concerned.

The architects were Messrs. Chorley, Connon, and Chorley, of Leeds, with Mr. Alexander Graham, of London, as consulting architect.

---

The foundation-stone has been laid of the new workhouse infirmary at Otley, near Bradford. It is to accommodate 70 patients. It is to be erected in three blocks, two for patients and one for administration. A glass corridor will connect the buildings, which are estimated to cost £8,571, which equals about £122 per bed.

## Figures and Tables

**Figure f1:**
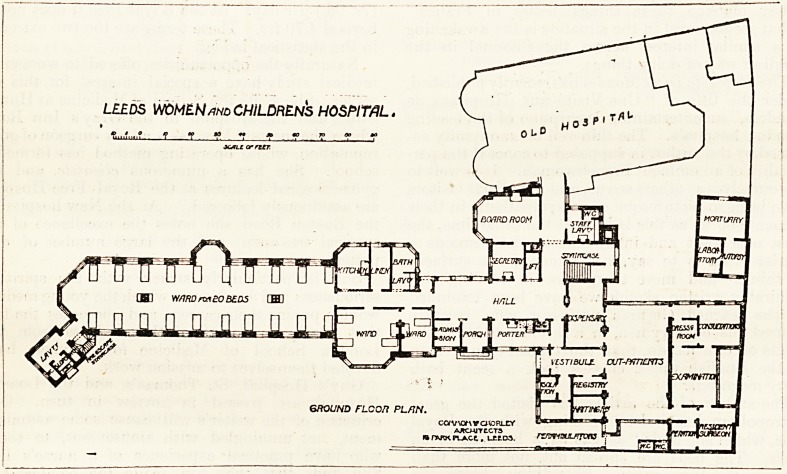


**Figure f2:**
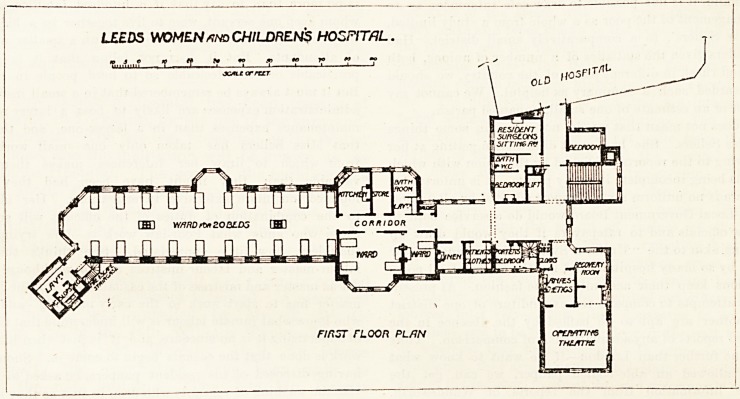


**Figure f3:**